# Perceived effectiveness rating scales applied to insomnia help-seeking messages for middle-aged Japanese people: a validity and reliability study

**DOI:** 10.1186/s12199-017-0676-x

**Published:** 2017-09-29

**Authors:** Machi Suka, Takashi Yamauchi, Hiroyuki Yanagisawa

**Affiliations:** Department of Public Health and Environmental Medicine, The Jikei University School of Medicine, 3-25-8 Nishi-Shimbashi, Minato-ku, Tokyo 105-8461 Japan

**Keywords:** Health message, Perceived effectiveness, Rating scale, Reliability, Validity, Japan

## Abstract

**Background:**

Communicating health messages is an important way to influence people’s behaviors towards health issues. Providers need to incorporate audience’s perspective to design more persuasive messages. This study aimed to develop rating scales for measuring audience’s perception of effectiveness of health messages in Japanese people.

**Methods:**

The comprehensibility scale including five items and the persuasiveness scale including seven items were designed based on literature review. A cross-sectional web-based survey was conducted among Japanese adults aged 35–45 years to assess the reliability and validity of the scales. Participants were asked to rate a text message that encouraged help-seeking intention for insomnia. All scale items were scored on a 1-to-5 point Likert scale, and they were averaged to produce an overall score for each scale.

**Results:**

Explanatory factor analysis revealed a two-factor solution that agreed with the comprehensibility and persuasiveness scales, respectively. Correlation coefficients between each set of items ranged between 0.63–0.87 for the comprehensibility scale and 0.37–0.76 for the persuasiveness scale. Cronbach alpha (0.88) indicated satisfactory internal consistency of the set of items. The mean (SD) of the comprehensibility and persuasiveness scores were 3.70 (0.82) and 3.15 (0.61), respectively, without ceiling or floor effects. These scores were significantly associated with intended future use of the message. The proportion of participants who reported a positive help-seeking intention for insomnia was significantly higher in the higher score groups for both scales. Multiple logistic regression analysis showed that the comprehensibility and persuasiveness scores were significantly associated with the help-seeking intention for insomnia.

**Conclusion:**

The proposed rating scales exhibited adequate reliability and validity for measuring the comprehensibility and persuasiveness of insomnia health-seeking message in middle-aged Japanese people. Further studies are needed to confirm the generalizability of the results, but these scales may be useful for pretesting a health message with audience members to make it more acceptable and persuasive to the intended audience.

**Trial registration:**

Not applicable; this is not a report of intervention trial.

**Electronic supplementary material:**

The online version of this article (10.1186/s12199-017-0676-x) contains supplementary material, which is available to authorized users.

## Backgrounds

Communicating health messages is an important way to influence people’s behaviors towards health issues [1–3]. The effect of health communication intervention depends on whether the message is effective or not [4]. Effective means that the message reaches the intended audience and delivers relevant, accurate, accessible, and understandable information, and it has to be acceptable and persuasive to the intended audience [1, 3, 5].

Most health communication comes to lay people from health professionals [6]. It is reasonable to expect discrepancies between messages sent and received, which may reduce the effectiveness of health communication intervention. Providers need to incorporate audience’s perspective to develop more effective massages [1, 2, 7]. A meta-analysis revealed that perceived message effectiveness is substantially correlated with actual effectiveness [8]. Pretesting a health message with audience members is recommended to know perceived message effectiveness prior to intervention implementation. It will help make refinements to enhance the effectiveness of health communication intervention [5].

A variety of methods have been proposed to measure perceived message effectiveness or perceived argument strength [9]. However, there is no measure of perceived message effectiveness available in Japan. Japanese people have their own unique language, custom, and culture. In order to facilitate improvements in health communication practices, a standard measure of perceived message effectiveness needs to be developed for Japanese people. This study aimed to develop rating scales for measuring audience’s perception of effectiveness of health messages in Japanese people. The following methods can be used to test health messages: focus groups, individual interviews, interviewer-administered questionnaires, and self-administered questionnaires [5]. Focus groups and individual interviews are often used for concept testing, but small sample sizes make it difficult to generalize their findings. Interviewer-administered questionnaires are more labor-intensive, time-consuming, and expensive compared with self-administered questionnaires. Therefore, our rating scales were designed to be self-administered by paper-and-pencil or via the Internet.

## Methods

We launched a research project to develop effective health communication interventions for encouraging help-seeking in people at risk of suicide. As the first step in the research project, this study attempted to develop a tool for pretesting health messages with audience members. The study protocol was approved by the ethics committees of the Jikei University School of Medicine and has been conducted in accordance with the Ethical Guidelines for Medical and Health Research Involving Human Subjects by the Japanese Government.

### Participants

A cross-sectional web-based survey was conducted in December 2016 among 1000 Japanese adults aged 35–45 years. Participants in the survey were recruited from an online research panel of a leading research company in Japan (Cross Marketing Inc., Tokyo, Japan). Recruitment emails were sent to 23,599 randomly selected eligible registrants. Medical professionals were excluded through a prescreening process. Applicants for participation in the survey were accepted in the order of receipt until the number of participants reached the quotas for gender, area, and K6 score (0–4 and 5+ points) [10]. All participants voluntarily agreed to participate in the survey after reading a description of the purpose and procedure of the survey. Consent to participate was implied by the completion and submission of the survey.

Of the 1000 respondents, 600 randomly selected people were asked to rate a text message that encouraged help-seeking intention for insomnia. Their responses were analyzed in this study. Table 1 shows the characteristics of the study participants. According to the national census [11], the percentage of the Japanese population aged 35–44 years with university degrees were 22.0% in 2010, considerably lower than that of this study (42.8%), whereas the percentages of married and employed population were 65.4 and 75.2%, respectively, in 2015, almost equal to that of this study (58.3 and 74.2%, respectively).

**Table 1:** Characteristics of the study participants

		*N*	
Gender	Male	300	50.0%
	Female	300	50.0%
Age	Mean (SD)	40.6	(3.0)
Education	Compulsory education/high school	176	29.3%
	Junior college/vocational school	167	27.8%
	University or higher	257	42.8%
Marital status	Married	350	58.3%
	Unmarried	221	36.8%
	Divorced/widowed	29	4.8%
Occupation	Full-time job	358	59.7%
	Temporary or part-time job	87	14.5%
	No occupation	155	25.8%
Household income	< 2.0 million yen^a^	76	12.7%
	2.0–3.9 million	120	20.0%
	4.0–5.9 million	173	28.8%
	6.0–7.9 million	112	18.7%
	8.0–9.9 million	58	9.7%
	10.0+ million	55	9.2%
	Missing	6	1.0%
Medical condition	Any disease	191	31.8%

^a^1 million yen was about 9000 US dollars at the time of the survey

### Measures

**Fig. 1:**
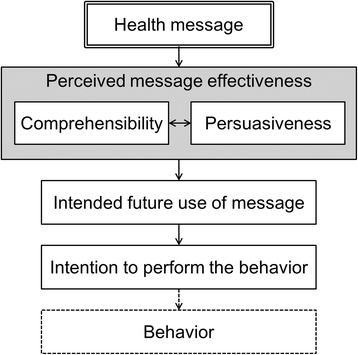
Conceptual framework of this study

Figure 1 shows the conceptual framework of this study. The study participants were asked to answer questions pertaining to perceived message effectiveness, intended future use of message, help-seeking intention for insomnia, and sociodemographic characteristics. The web questionnaire forms presented the questions one by one through the operation of a “Next” button. Respondents answered one question per page and could not go back to the previous page. The components of the questionnaire relevant to this study are detailed below.

### Message

A text message that encouraged help-seeking intention for insomnia was used by way of an example to assess the reliability and validity of the scales. The main message was “Sleeplessness lasting more than two weeks is a sign of depression. Talk with your primary care doctor,” which was derived from the Sleep Campaign launched by the Cabinet Office of the Japanese Government in 2010. In addition to the original version, two modified versions were prepared by adding a brief description of clinical depression. No significant differences were found between the original and modified messages in participants’ assessment (perceived effectiveness and intended future use). Therefore, the responses were lumped together in this study.

### Perceived message effectiveness

Perceived effectiveness can be defined as the subjective likelihood that a message will have a persuasive impact. Reading a message is the first step of the persuasion process. If recipients find difficulty in reading and understanding the message, it is unlikely to have a persuasive impact. Therefore, two rating scales were prepared for measuring comprehensibility and persuasiveness, respectively. Five items pertaining to comprehensibility and seven items pertaining to persuasiveness were derived from the literature [12–14].The questionnaire written in Japanese is shown in Additional file 1.

The Consumer Information Rating Form developed by Krass and colleagues [12, 13] contains three subscales: comprehensibility, utility, and design quality. In accordance with the comprehensibility subscale, the five items of the comprehensibility scale asked how easy or hard the information is to (1) read, (2) understand, (3) remember, (4) locate important information, and (5) keep for future reference. Response options were from 1 (very hard) to 5 (very easy). All item scores (range 1–5 points) were averaged to produce an overall score (i.e., comprehensibility score).

The Perceived Argument Strength Scale developed by Zhao and colleagues [14] contains seven components of positive perception of health messages: believability, convincingness, importance, confidence, friends, positive thoughts, and agreement. In accordance with these components, the seven items of the persuasiveness scale asked to what extent they agree or disagree that the information is (1) believable, (2) convincing, (3) important to me, (4) help me feel confident about how best to do, (5) would help my family and friends, (6) put thoughts in my mind about wanting to do, and (7) agreeable. Response options were from 1 (strongly disagree) to 5 (strongly agree). All item scores (range 1–5 points) were averaged to produce an overall score (i.e., persuasiveness score).

### Intended future use of message

Future use of message was measured by asking participants “If you saw the information in a newspaper or magazine, how likely would you use it?” Participants answered the question on a five-point scale ranging from 1 (very unlikely) to 5 (very likely).

### Help-seeking intention for insomnia

Help-seeking intention was measured using vignette methodology [15]. Participants were presented with a vignette describing a man (or woman) with insomnia and were then asked: “If you had health problems right now like Mr. A (or Ms. A), would you seek professional help?”

The description of the vignette was as follows:

- Mr. A (Ms. A) is a 40-year-old office worker. He (She) wake up frequently at night, so cannot sleep well despite being tired every night for more than 2 weeks. Accordingly, he (she) cannot go about his (her) work.

Participants answered the question on a four-point scale (certainly yes/probably yes/probably not/certainly not). Those who gave affirmative answers (certainly yes and probably yes) were counted as having a positive help-seeking intention.

### Statistical analysis

All statistical analyses were performed using the SAS ver. 9.4 (SAS Institute, Cary, NC, USA). Significant levels were set at *p* < 0.05.

### Reliability

Explanatory factor analysis with promax rotation was performed to determine the factor structure of the scales. Factor loadings of ≥ 0.4 were considered to be appropriate. Internal consistency was assessed by Cronbach alpha, where a value of ≥ 0.7 was considered satisfactory [16].

### Validity

Criterion validity was evaluated by the associations between the overall scale score and intended future use of the message. Construct validity was evaluated by the associations between the overall scale score (and each item score) and help-seeking intention for insomnia. One-way analysis of variance was used for comparing group means. Cochran-Armitage test was used for testing a linear trend in binomial proportions. Multiple logistic regression analysis was conducted to determine the association between the comprehensibility and persuasiveness scores and help-seeking intention for insomnia. Odds ratios (ORs) with 95% confidence intervals (CIs) for help-seeking intention for insomnia were calculated per 1-point increase in the comprehensibility and persuasiveness scores, respectively, with and without adjustment for underlying help-seeking intention for insomnia. Note that we had preliminarily confirmed that no demographic factors were significantly associated with help-seeking intention for insomnia.

## Results

### Reliability

Table 2 shows the factor structure of the comprehensibility and persuasiveness scales. Correlation coefficients between each set of items ranged between 0.63–0.87 for the comprehensibility scale and 0.37–0.76 for the persuasiveness scale. The initial factor solution indicated two factors with eigenvalues of 4.78 and 2.57, respectively, which jointly accounted for 102% of the total variance. The promax rotation indicated that all five items for the comprehensibility scale loaded on the second factor and that all seven items for the persuasiveness scale loaded on the first factor.

**Table 2:** Factor structure of the comprehensibility and persuasiveness scales

	Item score		Factor loadings		Communality
	Mean	SD	Factor 1	Factor 2	
(A) Comprehensibility scale					
1) Read	3.86	0.96	− 0.07	0.86	0.71
2) Understand	3.80	0.95	− 0.07	0.93	0.84
3) Remember	3.57	0.96	0.01	0.84	0.71
4) Locate	3.60	0.89	0.00	0.87	0.74
5) Keep	3.67	0.89	0.13	0.76	0.67
(B) Persuasiveness scale					
1) Believable	3.34	0.77	0.68	0.13	0.60
2) Convincing	3.26	0.78	0.72	0.08	0.62
3) Important	2.94	0.86	0.66	− 0.09	0.42
4) Confident	2.95	0.83	0.77	− 0.08	0.60
5) Friends	3.10	0.87	0.72	− 0.04	0.53
6) Positive thoughts	3.04	0.78	0.73	− 0.05	0.48
7) Agreeable	3.37	0.71	0.59	0.14	0.45

Comprehensibility scale items were rated on a scale of 1 (very hard) to 5 (very easy). Persuasiveness scale items were rated on a scale of 1 (strongly disagree) to 5 (strongly agree)

Table 3 shows the internal consistency of the comprehensibility and persuasiveness scales. The comprehensibility and persuasiveness scores approximated to a normal distribution with no ceiling or floor effects. These scores had a significant but weak correlation (*γ* = 0.27, *p* < 0.001). Cronbach alpha and corrected item-total correlation indicated satisfactory internal consistency of the set of items.

**Table 3:** Internal consistency of the comprehensibility and persuasiveness scales

	Overall scale score		Internal consistency among all items		Internal consistency among each scale items	
	Mean	SD	Cronbach alpha	Item-total correlation	Cronbach alpha	Item-total correlation
(A) Comprehensibility scale	3.70	0.82	0.88		0.93	
1) Read				0.54		0.80
2) Understand				0.58		0.87
3) Remember				0.57		0.81
4) Locate				0.59		0.83
5) Keep				0.65		0.77
(B) Persuasiveness scale	3.15	0.61			0.88	
1) Believable				0.66		0.71
2) Convincing				0.64		0.73
3) Important				0.45		0.60
4) Confident				0.55		0.71
5) Friends				0.53		0.67
6) Positive thoughts				0.51		0.65
7) Agreeable				0.59		0.62

Overall scale scores were calculated as the average of the scale items scored on a 1-to-5 point Likert scale. Internal consistency was assessed among all items and also among each scale items

### Validity

Table 4 shows the association between the overall scale score and intended future use of the message. The comprehensibility and persuasiveness scores were significantly associated with the intended future use of the message.

**Table 4:** Association between the overall scale score and intended future use of the message

	Intended future use of the message					
	1 (very unlikely)	2 (unlikely)	3 (neutral)	4 (likely)	5 (very likely)	*p*
Number of respondents	42	99	349	95	15	
Comprehensibility score	3.79 (1.14)	3.84 (0.76)	3.53 (0.74)	3.96 (0.84)	4.80 (0.54)	< 0.001
Persuasiveness score	2.53 (0.94)	2.95 (0.63)	3.14 (0.46)	3.52 (0.45)	4.05 (0.90)	< 0.001

Values were mean (SD). One-way analysis of variance was performed to identify significant differences

After reading the message, 287 participants (47.8%) reported having a positive help-seeking intention for insomnia. Table 5 shows the association between the overall scale score and help-seeking intention for insomnia. The proportion of participants having a positive help-seeking intention for insomnia was significantly higher in higher-score groups for both scales. Table 6 shows the association between each item score and help-seeking intention for insomnia. All scale items were significantly associated with the help-seeking intention for insomnia.

**Table 5:** Association between the overall scale score and help-seeking intention for insomnia

		Overall scale score					
		≤ 1.0	1.1–2.0	2.1–3.0	3.1–4.0	4.1–5.0	Trend *p*
Comprehensibility scale	*N*	5	7	191	227	170	0.009
	*n*	1	4	81	106	95	
		20.0%	57.1%	42.4%	46.7%	55.9%	
Persuasiveness scale	*N*	3	27	290	252	28	< 0.001
	*n*	0	4	99	160	24	
		0.0%	14.8%	34.1%	63.5%	85.7%	

Cochran-Armitage test was performed to identify a significant linear trend

*N* number of respondents, *n* number of people having a positive help-seeking intention for insomnia among the respondents

**Table 6:** Association between each item score and help-seeking intention for insomnia

		Item score					
		1	2	3	4	5	Trend *p*
(A) Comprehensibility scale							
1) Read	*N*	7	24	209	169	191	0.023
	*n*	1	13	87	85	101	
		14.3%	54.2%	41.6%	50.3%	52.9%	
2) Understand	*N*	7	32	205	185	171	0.040
	*n*	1	21	79	95	91	
		14.3%	65.6%	38.5%	51.4%	53.2%	
3) Remember	*N*	11	48	250	168	123	< 0.001
	*n*	3	23	96	92	73	
		27.3%	47.9%	38.4%	54.8%	59.3%	
4) Locate	*N*	8	26	280	170	116	< 0.001
	*n*	3	12	111	89	72	
		37.5%	46.2%	39.6%	52.4%	62.1%	
5) Keep	*N*	9	24	245	203	119	< 0.001
	*n*	1	14	92	99	81	
		11.1%	58.3%	37.6%	48.8%	68.1%	
(B) Persuasiveness scale							
1) Believable	*N*	13	43	292	226	26	< 0.001
	*n*	3	11	108	145	20	
		23.1%	25.6%	37.0%	64.2%	76.9%	
2) Convincing	*N*	15	57	308	199	21	< 0.001
	*n*	3	17	128	120	19	
		20.0%	29.8%	41.6%	60.3%	90.5%	
3) Important	*N*	35	115	320	110	20	< 0.001
	*n*	14	39	145	71	18	
		40.0%	33.9%	45.3%	64.5%	90.0%	
4) Confident	*N*	37	95	344	107	17	< 0.001
	*n*	10	34	151	77	15	
		27.0%	35.8%	43.9%	72.0%	88.2%	
5) Friends	*N*	32	80	310	155	23	< 0.001
	*n*	11	28	132	96	20	
		34.4%	35.0%	42.6%	61.9%	87.0%	
6) Positive thoughts	*N*	31	56	386	108	19	< 0.001
	*n*	6	12	169	82	18	
		19.4%	21.4%	43.8%	75.9%	94.7%	
7) Agreeable	*N*	9	20	346	192	33	< 0.001
	*n*	0	7	131	120	29	
		0.0%	35.0%	37.9%	62.5%	87.9%	

Cochran-Armitage test was performed to identify a significant linear trend

*N* number of respondents, *n* number of people having a positive help-seeking intention for insomnia among the respondents

As for the help-seeking intention for insomnia, crude ORs (95% CIs) per 1-point increase in the comprehensibility and persuasiveness scores were 1.47 (1.21–1.80) and 4.23 (2.96–6.03), respectively. Adjusted ORs (95% CIs) per 1-point increase in the comprehensibility and persuasiveness scores were 1.38 (1.02–1.85) and 3.40 (2.13–5.43), respectively.

## Discussion

We developed rating scales for measuring audience’s perception of the effectiveness of health messages in Japanese people. Health communication is essential for public health [1–3]. As stated in the practical guidelines on health communication [5], providers need to test and refine their messages to implement effective health communication interventions. There have been few attempts to propose a standard measure of perceived message effectiveness that can be used across various contexts [14]. Also, less attention has been paid to the effectiveness of health messages in Japan, where almost 100% of the population are enrolled in compulsory grades and can read and write the Japanese language. The results of this study revealed that the proposed rating scales had adequate reliability and validity for measuring the comprehensibility and persuasiveness of insomnia health-seeking message in middle-aged Japanese people. These scales may be useful for pretesting a health message with audience members to make it more acceptable and persuasive to the intended audience.

The comprehensibility and persuasiveness scales were proven reliable by Cronbach alpha along with explanatory factor analysis. The criterion validity was confirmed, as the overall scale scores were significantly associated with intended future use of the message. The construct validity was confirmed, as the overall scale scores were significantly associated with help-seeking intention for insomnia. From the point of view of a provider, measures of perceived message effectiveness are expected to predict actual effectiveness [8]. The message used in this study was designed to encourage help-seeking intention for insomnia. The significant association with help-seeking intention for insomnia suggested that the rating scales can be a useful predictor that indicates the likely effect of health message with reasonable precision.

The results of this study revealed that the comprehensibility and persuasiveness scales are reliable and valid enough for practical use. However, in general, caution should be exercised in measuring and interpreting perceived message effectiveness. Non-target audience members tend to underestimate perceived message effectiveness compared with target audience members [17]. Researchers need to enroll the intended audience in the assessment of message to obtain unbiased estimates. The measures of perceived message effectiveness may predict future behavior change to some extent, but do not directly measure potential behavior change [9]. A health message is finally judged effective if it produces a positive change in the variable that it was designed to change. In most cases, providers aim at changing people’s behaviors towards health issues. Researchers should be careful in using the persuasive effectiveness rating scales and interpreting their measurement results. It may be recommended to assess actual changes in audience’s behaviors to confirm the effect of health communication intervention.

In this study, we could not compare differences between two or more health messages or differences between original and revised versions of a health message. Another study is now in progress to demonstrate the usefulness of our rating scales. We will compare different health messages and examine the association between message features and perceived message effectiveness. We will be able to propose a practical method for developing effective health messages using the comprehensibility and persuasiveness scales.

This study provides evidence for the reliability and validity of the comprehensibility and persuasiveness scales. On the contrary, it has a number of potential limitations. First, the web-based survey was self-administered, so that the accuracy of responses would depend on participants’ understanding of the questions and their motivation to answer questions accurately. The understandability of the wording of items was checked prior to the survey. The use of the Internet and the provision of anonymity would be expected to elicit more truthful responses, by minimizing social desirability pressures [18]. However, it is almost impossible to eliminate the information bias completely. Second, the study participants were selected from a nationwide panel of a research company. As described in the “Methods” section, the study participants included highly educated people twice as many as in the Japanese population. Although we confirmed that the distribution of HLS-14 scores (measurements of generic health literacy) in the study participants is quite similar to that obtained from our previous paper-based survey in a Japanese healthcare facility [19], the selection bias may have influenced the results to some extent. Third, in the absence of a gold standard, the criterion validity was evaluated by the association with a criterion variable that assessed from our original single question. Little work has been done on the conceptual meaning of perceived effectiveness, so that its measures were inconsistent across studies [9]. Further studies are required to examine whether our rating scales perform better than others to acknowledge as a standard measure of perceived message effectiveness in Japan.

## Conclusion

The proposed rating scales exhibited adequate reliability and validity for measuring the comprehensibility and persuasiveness of insomnia health-seeking message in middle-aged Japanese people. Further studies are needed to confirm the generalizability of the results, but these scales may be useful for pretesting a health message with audience members to make it more acceptable and persuasive to the intended audience.

## Additional file

Additional file 1: Rating scales for measuring audience’s perception of effectiveness of health messages in Japanese people (DOC 61 kb)
