# Supplementary material for: Perceived effectiveness rating scales applied to insomnia help-seeking messages for middle-aged Japanese people: a validity and reliability study
Source: Environ Health Prev Med. 2017 Sep 29;22:69. doi: 10.1186/s12199-017-0676-x (PMC5664822; doi:10.1186/s12199-017-0676-x)
Supplement: Additional file 1: — Rating scales for measuring audience’s perception of effectiveness of health messages in Japanese people (DOC 61 kb) [file 12199_2017_676_MOESM1_ESM.doc]

Additional file 1: Rating scales for measuring audience’s perception of effectiveness of health messages in Japanese people

Comprehensibility scale

この文章について、あなたが以下のように行動することは易しいと思いますか。

How easy or hard would you say the information is to…

|  | とても  難しい  very hard | やや  難しい  quite hard | どちらとも  言えない  in between | やや  易しい  quite easy | とても  易しい  very easy |
| --- | --- | --- | --- | --- | --- |
| 1. 読む   read | 1 | 2 | 3 | 4 | 5 |
| 1. 理解する   understand | 1 | 2 | 3 | 4 | 5 |
| 1. 記憶する   remember | 1 | 2 | 3 | 4 | 5 |
| 1. 重要な情報を指し示す   locate important information | 1 | 2 | 3 | 4 | 5 |
| 1. 今後の参考にする   Keep for future reference | 1 | 2 | 3 | 4 | 5 |

Persuasiveness scale

この文章について、あなたはどのように考えましたか。

To what extent do you agree or disagree that the information is…

|  | 全くそう  でない  strongly disagree | あまりそう  でない  disagree | どちらとも  言えない  unsure | まあ  そうだ  agree | 全く  そうだ  strongly agree |
| --- | --- | --- | --- | --- | --- |
| 1. 信用できる   believable | 1 | 2 | 3 | 4 | 5 |
| 1. 説得力がある   convincing | 1 | 2 | 3 | 4 | 5 |
| 1. 自分にとって重要だ   important to me | 1 | 2 | 3 | 4 | 5 |
| 1. 何が最も良い方法かを確信させた   help me feel confident about how best to do | 1 | 2 | 3 | 4 | 5 |
| 1. 家族や友人の役に立つだろう   would help my family and friends | 1 | 2 | 3 | 4 | 5 |
| 1. 実行したいと思った   put thoughts in my mind about wanting to do | 1 | 2 | 3 | 4 | 5 |
| 1. 賛成できる   agreeable | 1 | 2 | 3 | 4 | 5 |
